# Graph Convolutional Network for 3D Object Pose Estimation in a Point Cloud

**DOI:** 10.3390/s22218166

**Published:** 2022-10-25

**Authors:** Tae-Won Jung, Chi-Seo Jeong, In-Seon Kim, Min-Su Yu, Soon-Chul Kwon, Kye-Dong Jung

**Affiliations:** 1 Department of Immersive Content Convergence, Kwangwoon University, 20 Kwangwoon-ro, Nowon-gu, Seoul 01897, Korea; 2 Department of Smart Convergence, Kwangwoon University, 20 Kwangwoon-ro, Nowon-gu, Seoul 01897, Korea; 3 Ingenium College of Liberal Arts, Kwangwoon University, 20 Kwangwoon-ro, Nowon-gu, Seoul 01897, Korea

**Keywords:** graph neural network, graph convolutional network, three-dimensional object detection, three-dimensional object pose estimation, three-dimensional point cloud, one-stage detection method

## Abstract

Graph Neural Networks (GNNs) are neural networks that learn the representation of nodes and associated edges that connect it to every other node while maintaining graph representation. Graph Convolutional Neural Networks (GCNs), as a representative method in GNNs, in the context of computer vision, utilize conventional Convolutional Neural Networks (CNNs) to process data supported by graphs. This paper proposes a one-stage GCN approach for 3D object detection and poses estimation by structuring non-linearly distributed points of a graph. Our network provides the required details to analyze, generate and estimate bounding boxes by spatially structuring the input data into graphs. Our method proposes a keypoint attention mechanism that aggregates the relative features between each point to estimate the category and pose of the object to which the vertices of the graph belong, and also designs nine degrees of freedom of multi-object pose estimation. In addition, to avoid gimbal lock in 3D space, we use quaternion rotation, instead of Euler angle. Experimental results showed that memory usage and efficiency could be improved by aggregating point features from the point cloud and their neighbors in a graph structure. Overall, the system achieved comparable performance against state-of-the-art systems.

## 1. Introduction

Computer vision is a research field in computer science that studies the components associated with digital images or videos. Recently, deep learning-based 3D object detection methods, using 3D sensors, have been actively proposed in various fields, such as autonomous driving and robotics [1]. For effective end-to-end systems for deploying 3D object detection methods, state-of-the-art studies are being conducted on CNN, Recurrent Neural Networks (RNNs), and cloud computing processing for the integration of long-term short-term memory, and distributed systems, as well as parallelization within and between networks [2,3]. With the development of 3D information acquisition hardware-assisted technologies, there are various types of 3D scanners, LiDARs, and RGB-D image sensors for obtaining scaled information [4]. The 3D sensor uses infrared rays to determine the depth information between the object and the sensor by measuring the change in the waveform of the wavelength reflected by the object or the time difference. The depth and color information of objects in 3D can be obtained and converted spatially to construct a point cloud, which is a 3D data structure [5]. The characteristics of 3D data contain more information than those of 2D. This enables the networks to easily distinguish occluded objects. Therefore, 3D data acquired from small IT devices, have been applied to 3D shape classification, such as object detection and tracking, point cloud segmentation, and point cloud processing [6].

Point clouds are used in many fields, such as in the military, education, medical care, and architecture [7], and deep learning is being applied to efficiently process them. There are two methods for processing a point cloud by deep learning: a method that implies spatial information and a method that directly uses point information [8]. A 3D CNN is a typical example of a method for processing implied spatial information [9]. CNN is a deep learning model that is widely used in the image field and can efficiently collect and process image features. However, in the case of 3D models, a large amount of computation occurs, owing to the application of CNN filters when projected spatial features are implied [10]. Additionally, the loss of 3D coordinate information of a point occurs during the implication process. Defining the best point cloud representation for deep learning is not straightforward and remains an open problem [11,12,13]. To address this problem, several studies have used GNN for the classification and semantic segmentation of point clouds [6,14,15,16]. A Point-GNN, which expresses the point cloud as a graph, has a wide receptive field while directly processing point information [17]. The recent development of GCN, which is a representative method of GNN, suggests that graph representation can provide improved functions for irregular point cloud processing [18].

Our previous study [19] addressed the novel VPS (Visual Positioning System) to determine the user’s position in an indoor space without GPS by matching the point cloud pose database, which is addressed by pre-defined voxel indexes and object pose estimation. The network in this system improved Point-GNN [17], and used MLP (Multi-Layer Perceptron) to estimate the pose of 3D objects, and proposed 3D IoU (Intersection Over Union) using Euler rotation. The Euler method has been used in algorithms related to several 3D spaces. The conventional method was to calculate two 3D bounding boxes, IoU in seven DOF (Degrees of Freedom) in one rotation (centered on the *z*-axis) using a bird’s eye view. However, we designed the nine DOF estimation of an object pose, which improved the limits of the seven DOF pose estimation. We adjusted the new network structure to improve performance, such as accuracy and real-time operation. 

In this paper, we propose a 3D point cloud object detection and pose estimation method based on a graph convolution network and the keypoint attention mechanism to aggregate the features of neighboring points. The partial concept of our method is inspired by Point-GNN [17]. The proposed method represents the point cloud as its graph, processes the structured adjacency matrix and feature matrix as inputs, and outputs the class, the bounding box, the size of the object, and the pose of the object. Their outputs are estimated, based on the vertices. All processes are dealt with in one-stage, while detecting multiple objects. Our method computes a rotated 3D IoU around all axes with nine DOF (three coordinates, three translations, and three rotations) for pose estimation in a 3D space. Additionally, to overcome the gimbal lock caused by Euler rotation in 3D space, we use the quaternion rotation. Our proposed method is evaluated on the RGB-D Dataset 7-Scene and demonstrates its potential in a 3D spatial processing domain using GCN.

The contributions of this study can be summarized as follows:

- We propose a one-stage object detection and pose estimation approach using a graph convolutional network based on point cloud.
- We design a point cloud-based graph convolutional network with a keypoint attention mechanism.
- In the RGB-D Dataset 7-Scene, 3D objects are estimated with nine DOF, and rotation error is overcome to achieve comparable performance with state-of-the-art systems.

The remainder of this paper is organized as follows. Section 2 provides a brief overview of the work, and Section 3 details the system architecture and the proposed methodology. Section 4 presents the experiments and performance evaluations. Section 5 proceeds with an analysis of the obtained results, and Section 6 summarizes the conclusions. The code is available at github.com/onomcanbot/Pointcloud-GCN (accessed on 16 August 2022).

## 2. Related Work

### 2.1. Graph Neural Network Models

CNNs have been successfully applied to many tasks, such as image classification, semantic segmentation, and machine translation; however, in this case, the data had to be expressed in a grid structure, which is difficult with data from many fields. Examples include 3D meshes, social networks, telecommunications networks, and biological networks. In addition, the loss of 3D coordinate information of a point occurs in the process of implying spatial features. For this reason, information on points, such as in PointNet, was structured as a set and processed directly [11]. As another method of structuring point clouds, the distribution of irregular points can be expressed as a graph structure. Attempts to extend the neural network to deal with graphs of various structures have continued, and the initial research attempted to deal with data of graph structures with recurrent neural networks (RNNs). 

GNN was introduced as a generalized version of the RNN, and innovative methods have been continuously proposed since then [20]. Conventionally, GNNs collect information from neighboring nodes, utilizing convolutional calculation, and the collected data is applied to the neural network. The GNN layers consist of a messaging and aggregation layer. From this perspective, there are GCN, GraphSAGE, and GAT.

Graph spectral filtering is defined as a Fourier domain that calculates the Eigen decomposition of the graph Laplacian, and performing such a convolution operation is a representative method. Through this operation, intense operations and nonspatially localized filters can be created. This method is specialized to reflect a more detailed structure and feature characteristics but has difficulty reflecting the characteristics of a new graph structure or a new node. Graph convolutional neural network (GCN) is a method of applying convolution to a graph approached from a spectral point of view. The GCNs have the same weighting value for both neighboring nodes and the node itself. There is no weight matrix in the GCN for the embedding value in the previous layer of its node. Nodes and neighboring nodes use the same weight matrix [21].

Graph spatial filtering refers to a method of directly applying a convolution operation to a graph and performing an operation on a spatially close neighbor group. A representative method is GraphSAGE [22]. In this work, the weight is determined by the concatenation, rather than the addition, of self-embedding and neighbor aggregation. As an aggregation function, Mean, Pool, and Sum functions can be used; normally the Mean method is utilized, which leverages the embedding values of neighboring nodes. This averaging function is also used in the neighboring aggregation. This approach is effective for very large-scale data. In graph attention (GAT) networks [23], the attention-based operation is efficient for data in a graph structure, and, by specifying different weights for neighbors, it can be applied to graph nodes with different degrees equal to the number of neighbors of the node. In GAT, the importance of the node feature is used to calculate the attention coefficient, like the attention mechanism, and normalized by softmax function. In addition, since this model can be directly applied to the inductive learning problem, it also generalizes to previously untrained graphs.

### 2.2. One-Stage and Two-Stage Object Detection Models

Generally, 3D detectors can be classified into two types. The one-stage detector simultaneously predicts regional proposal and classification problems for features learned from the input, whereas the two-stage detector uses the second-stage prediction to refine the first-stage regional proposal. The two-stage detector object detection region-based approach can be computationally expensive in mobile and wearable systems, with limited storage and computational memory. One-stage detectors are generally faster with a simple network architecture, whereas two-stage detectors often achieve higher accuracy in the additional stage. Therefore, instead of attempting to optimize the course of the region-based pipeline, several studies have used an integrated detection strategy.

According to recent trends, the precision of one-stage detectors [24,25] gradually approaches that of two-stage detectors. This motivated researchers to focus on developing the one-stage method and aim for high accuracy and speed. Point-GNN introduced an auto-registration mechanism that can align coordinates according to the features of the points to reduce the transformation variance of the GNN, and presented box merging and scoring operations to accurately combine the detection results at multi-points [17]. VoxelNet splits the point cloud into voxels and extracts the voxel features using the points in each voxel through the voxel feature encoding layer. Then, the feature-bearing voxels are integrated with local voxel features through 3D convolution, and a bounding box is generated using a region proposal network [8]. PointPillars enabled end-to-end training of 3D object detection networks using a new encoder that learns features in vertical columns of point clouds [26]. SECOND modified sparse convolution to extract features from sparse voxels efficiently [27]. Finally, SE-SSD achieved the highest average precision and very high efficiency in both 3D and Bird’s eye view detection in point cloud compared with all previous one- and two-stage detectors [28].

### 2.3. Three-Dimensional Intersection over Union

The IoU frequently referred to as Jaccard Coefficients is the value of the intersection of two sets divided by the union and is widely adopted as an evaluation metric for computer vision research, such as instance-based segmentation, semantic segmentation, object detection, and multi-object tracking in 2D and 3D. It measures the closeness of the predicted bounding box to the ground truth. The formal method used for autonomous driving datasets (e.g., [29]) is to project a 3D boundary box onto the ground and then find the intersection with the 2D projection polygon. The cross-volume is then estimated by multiplying the height of the 3D bounding box. This method applies to the KITTI benchmark, for example, but has two limitations. The first is that the 9 DOF of the box is limited to 7 DoF because the object must be on the same ground. The box has only yaw DOF, and the roll and pitch are not predicted. The second assumption was that the boxes were of the same height. However, in 3D object detection, the exact 3D IoU value of a 3D box can be calculated using a three-part algorithm [30]. Our method solves the problem of previous studies that calculate by fixing one or more axes by calculating a rotating box in 3D.

## 3. Proposed Method

**Figure 1:**
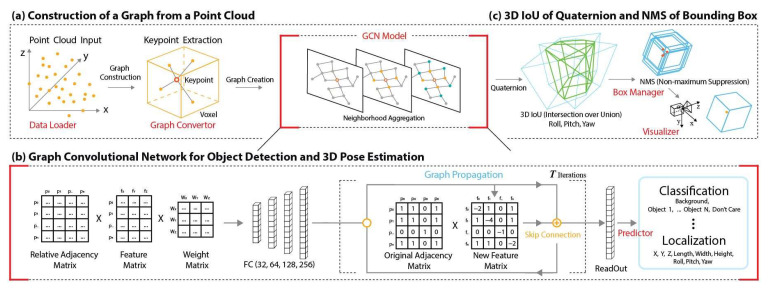
Proposed method with three components.

We propose a method for object detection and pose estimation in point clouds. As shown in Figure 1, the overall architecture comprises three components: (a) graph construction, (b) GCN of iterations, and (c) 3D IoU of quaternion and non-maximum suppression (NMS) of bounding box. Our architecture of the point cloud-based GCN model combines the keypoint attention mechanism, which can merge the neighboring features, with the main hierarchical types of graph convolution, skip connection, and readout.

### 3.1. Keypoint Extraction and Graph Matrix

#### 3.1.1. Keypoint Extraction for Graph Representation

Edge reconstruction-based techniques through image analysis include Delaunay triangulation and Voronoi Diagram. Delaunay triangulation refers to a division in which the minimum value of the interior angles of these triangles becomes the maximum when the space is divided by connecting points on the plane with a triangle. The Voronoi Diagram is a diagram in which the plane is divided, and it is a diagram in which the area is divided according to which point on the plane is closest to the seed point, which is the seed point given on the plane.

Firstly, we define a point cloud of *N* points in Euclidean space as set $V=\left\{v_{1},\;\cdots ,v_{N}\right\}$; $v_{i}=\left(x_{i},\;s_{i}\right)$ is a point with both the 3D coordinates, where $x_{i}$ is a point property in ${\mathbb{R}}^{k}$ domain, and the vector value of the state, $s_{i}\in \;{\mathbb{R}}^{k}$. Then, we construct a graph *G* = (*V*, *E*) using *V* as the vertex and connecting the points to the neighbors in a sphere with a fixed radius, and *E* connects points to the neighbor of a sphere with a fixed radius.

We refer to [17], and the fixed-radius near neighbors problem [31], where one wants to efficiently find all points given within an assigned fixed distance from a specified point in Euclidean space. Our method can efficiently solve the *O(n)* runtime complexity problem. Point clouds typically consist of tens of thousands to millions of points; thus, a graph structured with all points as vertices is extremely inefficient, such as in memory outcome. Therefore, down-sampled point clouds were used for graph construction. The space was divided by calculating the maximum value of the spatial coordinates of the point cloud, determining the size of the space, and dividing the space by the set voxel size. Since the number of graph vertices is determined according to the voxel size, it was set according to the size of the object to be detected. Formally, we called the graph vertices keypoints. The coordinates of each point cloud were divided by the voxel size, and the resulting quotient was used as an index to represent the position of the voxel. Based on the created voxel index, the final keypoint was determined through random extraction of points belonging to the same voxel. Keypoints were determined according to the order in which they were created. The extracted keypoints were connected through a sphere-based neighbor-proximity search and structured as a graph. The radius of the sphere, which is the extraction range, was set to a range that included the size of the object. A set of graph edges centered on each keypoint was generated, based on the coordinate information of the keypoint and the created index.

#### 3.1.2. Graph Matrix

**Figure 2:**
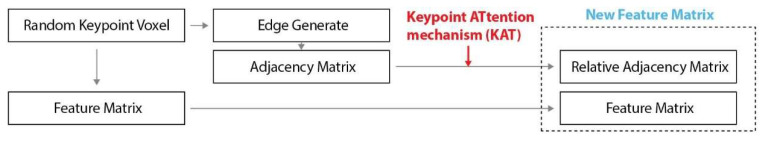
Keypoint extraction and conversion to graph matrices.

Figure 2 is the flow of the Graph Converting process for extracting keypoints of our method and generating graphs with keypoints as vertices. The point cloud, structured as a graph, is expressed in the form of a sparse matrix, because keypoints are connected according to a certain range, and not all keypoints are connected. The adjacency matrix is expressed in a form that does not connect itself when a loop does not exist. In this case, there is a problem, in that the feature of the vertex itself is not included in the feature matrix and the later matrix multiplication operation. To solve this problem, a general GCN creates an adjacency matrix that reflects the loop, including the vertex itself. When a vertex point includes its features, it creates a loop that connects itself to create an original adjacency matrix that is added to the existing edge list. The original adjacency matrix generated in this manner can indicate the connection state between points, but does not contain information about the relative distance from the vertex point. Our method is based on graph node attention. Graph attention networks (GATs) use attention to learn the relative weights between two connected nodes in message passing [22]. We propose the Keypoint Attention Mechanism (KAT), which introduces the concept of GAT, which includes different weights for each vertex. The KAT calculates the attention for node pairs i and j and uses the relative adjacency matrix to reflect the relative information between points in the GCN structure. The relative adjacency matrix is obtained through the difference between the adjacency matrix, representing the connection relationship and points in the degree matrix representing the number of edges of the points.

**Figure 3:**
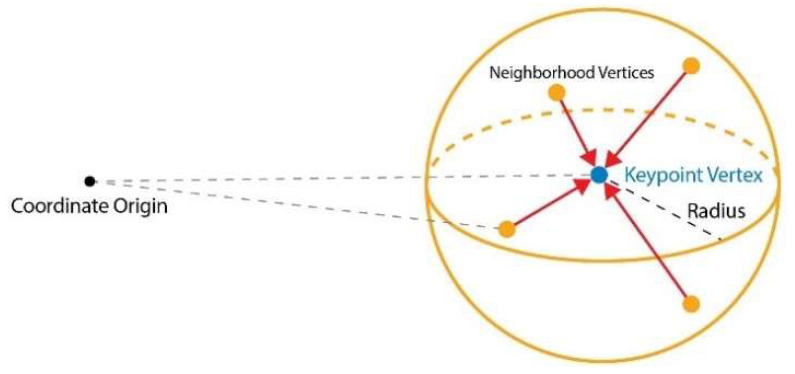
Using the keypoint attention mechanism, the coordinate features of the keypoint aggregate the features of the coordinates of the keypoint and the connected neighbor vertex.

In Figure 3, a negative number was taken in the relative adjacency matrix, the connection relation of the points was expressed as a positive number, and the number of edges was expressed as a negative number, which was used as the relative adjacency matrix. The relative adjacency matrix did not add the coordinate features (x,y,z) of the center point ($x_{p_{center}}$), unlike when calculating matrix multiplication with the feature matrix. It was expressed in relative coordinates between adjacent points $\left(x_{p_{k}}\right)$ by subtracting the number of neighboring points connected to the center point.

(1) $$\Sigma_{k=1}^{n}\left(x_{p_{k}}-x_{p_{center}}\right)$$

Equation (1), which modifies the state of a vertex using the state of a neighbor, is similar to each vector value in terms of direction and magnitude.

**Figure 4:**
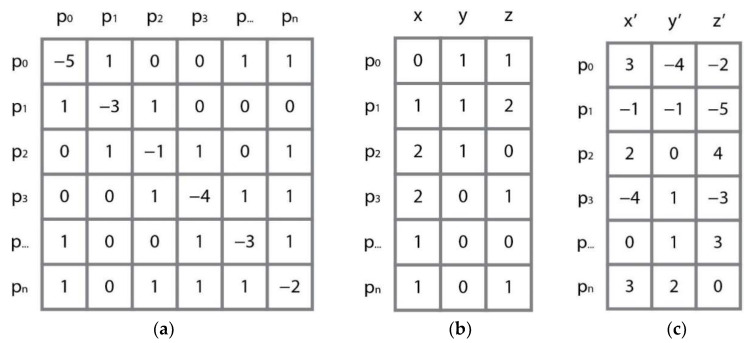
Matrix operations: (**a**) rows of the relative adjacency matrix; (**b**) the coordinate matrix; and (**c**) new feature matrix.

Figure 4 shows an example of an adjacency matrix configuration and operation with a coordinate matrix. The row of the relative adjacency matrix and the column of the coordinate matrix are multiplied to generate a new feature matrix that reflects the relative characteristics.

### 3.2. Graph Convolutional Network for Object Pose Estimation

#### 3.2.1. Convolutional Layer

The graph 𝒢(V, E) was defined as an ordered pair of sets V of vertices and E of edges, where the edge $e_{i,j}\in E$ was the connection between nodes $v_{i}\in V$ and $v_{j}\in V$. Since the connection between nodes was directional, $e_{i,\;j}$ and $e_{j,\;i}$ were different. Each node was also represented by a c-dimensional feature vector $v_{i}\in {\mathbb{R}}^{c}$. 

Given an input graph 𝒢, the graph convolution operation F aggregated the features of the *k* nodes in the neighbor $N^{\left(k\right)}\left(V\right)$ of the given node *v*. The convolution operation F aggregated function between adjacent nodes and updates the value of a given node (Equation (2)). It is a CNN concept in which convolutional filter aggregates surrounding pixels, aggregating the functions of graph neighbor nodes. GCNs apply the same weight value to different neighbors.

(2) $$F\left(G_{l},\;W_{l}\right)=Update\left(Aggregate\left(G_{l},\;W_{l}^{a}\right),\;W_{l}^{u}\right)\;G_{l+1}=F\left(G_{l},\;W_{l}\right)+G_{l}$$

The first process generated a value that reflected the relative information between points by multiplying the relative adjacency matrix generated in the preprocessing step and the coordinate feature matrix. By accumulating the unique features (RGB) of the corresponding values and keypoints, it passed through a multilayer neural network [32, 64, 128, 256] to generate high-dimensional features. The second process proceeded with the GCN operation, which multiplied the original adjacency matrix and feature matrix created in the previous process. The GCN operation was performed three times, and the information up to the 3rd-order adjacency of the graph was aggregated. In the third process, the aggregated information was passed through a multilayer neural network [256] and transformed into a value with the order between features removed. Finally, we predicted the class and box, based on the values from the previous step. Classes were passed through a multilayer neural network [64, 3] to generate predictions for each class. The corresponding value was later passed through softmax and used as the probability value of each class. The box indicated the number of classes.

#### 3.2.2. Skip Connection

**Figure 5:**
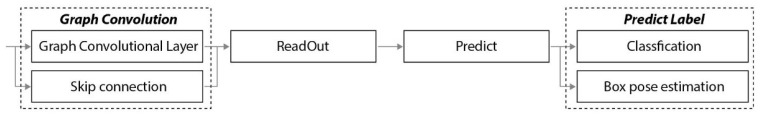
The flow of Convolutional Layer, Skip connection, ReadOut.

Figure 5 shows the flow of skip connection and readout in the GCN model. The skip connection adds previous features to the new features created through the graph convolution process. This solves the problem of features disappearing through differentiation in the backpropagation process, and the number of adjacent graphs increases as the number of connections increases. In our method, to use the information of the previous layer, we added the features of the original point by linking the information of the previous and next layers. This was performed so as to skip features from the contracting path to the expanding path to recover the spatial information lost during down-sampling. The expanding path recovered spatial information by merging skipped features at various resolution levels in the contracting path.

#### 3.2.3. Loss

Classification converted the input of the ground truth class into matrix data consisting of integer values using a one-hot encoding process. The predicted value was passed through softmax, as in $f{\left(s\right)}_{i}$ in Equation (3), to make it a value between 0 and 1 and then passed the log. The categorical cross-entropy error $f_{cls}$ of the class was calculated using the ground truth label and prediction so that the greater the uncertainty of the prediction, the greater the error. Cross entropy was calculated using the distribution between the predicted class and the actual class among keypoints with the actual ground truth $t_{i}$ and the predicted value $f{\left(s\right)}_{i}$ during training. Due to the nature of object detection, the distribution of the background class was larger than the distribution of the class of the object; therefore, it was difficult to efficiently learn when only the error was obtained, and thus cross-entropy was used. Equation (3) shows the calculation of categorical cross-entropy:

(3) $$f{\left(s\right)}_{i}=\frac{e^{S_{i}}}{\sum_{j}^{c}e^{S_{j}}}\;f_{cls}=-\Sigma_{i}^{c}\;t_{i}\;\log\left(f{\left(s\right)}_{i}\right)$$

For efficient learning, localization performed learning in the form of increasing box accuracy through the ground truth label, instead of the predicted value, while learning was in progress. Since the box must predict an accurate value, rather than a probability prediction, a Huber loss function that is not sensitive to outliers was used. The Huber loss function $f_{loc}$ uses the least square error (L2 error) when the absolute values of the ground truth $y_{i}$ and the prediction error ${\hat{y}}_{i}$ are less than δ and the least absolute deviations (L1 error) for the region greater than δ. By combining the L1 and L2 errors, differentiation is possible in all sections; therefore, it is easy for the backpropagation process. Equation (4) shows the Huber loss function calculation:

(4) $$f_{loc}=\left\{\begin{array}{c} \frac{1}{2}{\left(y_{i}-{\hat{y}}_{i}\right)}^{2}\;\;\;\;\;\;\;\;\;\;\;\;\;\;for\;\left|y_{i}-{\hat{y}}_{i}\right|\leq \delta \;\;\;\;\;\;\;\;\;\;\;\;\;\;\;\\ \delta \left|y_{i}-{\hat{y}}_{i}\right|-\frac{1}{2}\delta^{2}\;\;\;\;\;\;\;\;\;\;otherwise\;\;\;\;\;\;\;\;\;\;\;\;\;\;\;\;\;\;\;\;\; \end{array}\right.$$

### 3.3. Intersection over Union and Bounding Box

#### 3.3.1. Quaternion

Euler rotation defines ω, φ, and κ as angles that rotate in the clockwise direction, based on the *x*-, *y*-, and *z*-axes of the 3D coordinate system, respectively. When using the Euler angle, there is a case in which the two rotation axes overlap, and the gimbal-lock phenomenon occurs in which the directionality of one axis is lost. The proposed method solved the gimbal-lock problem. By introducing the concept of a quaternion complex number to express the rotation of each axis, the gimbal lock, the instability that occurs when the size of φ increases, and the increase in the amount of computation, were solved. A quaternion is a vector with four components: three are imaginary parts, and one is a real part. We defined $q=w+x_{i}+y_{j}+z_{k}$. $R_{q}$ in Equation (5) is a quaternion rotation matrix:

(5) $$R_{q}=\left[\begin{array}{c} \;\begin{array}{cccc} 1-2y^{2}-2z^{2} & 2xy-2wz & 2xz+2wy & 0\\ 2xy+2wz & 1-2x^{2}-2z^{2} & 2yz-2wx & 0\\ 2xz-2wy & 2yz+2wx & 1-2x^{2}-2y^{2} & 0\\ 0 & 0 & 0 & 1 \end{array}\;\; \end{array}\right]$$

#### 3.3.2. 3D Intersection over Union

**Figure 6:**
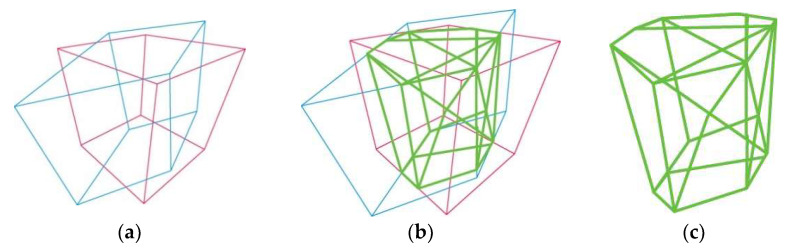
3D IoU with pink and blue lines boxes: (**a**) clipping the polygon to the box to calculate the intersection of each side; (**b**) calculating the intersection volume using the convex hull algorithm of all intersections (green); (**c**) 3D IoU.

The exact 3D IoU value of a 3D box can be calculated using a three-part algorithm. First, the Sutherland–Hodgman polygon-clipping algorithm computed the intersection points between the faces of the two boxes. Then, we estimated intersection volumes after the intersected points of the polygons were decided to form planes of intersections and polygons in the new coordinate system. The edges of the plane extended to virtual infinity to cut the polygon concerning the plane. It was determined whether that edge intersected the face of the axis alignment box. Second, the intersecting volume used the convex hull algorithm for all truncated polygons. Finally, the 3D IoU value was determined by unifying all estimated intersecting polygons in the world coordination system. Figure 6a shows polygon clipping. The volume of the intersection was calculated using the convex hull of all truncated polygons, as shown in Figure 6b. Third, the 3D IoU was calculated based on the intersection and binding volumes of the two boxes, as shown in Figure 6c.

#### 3.3.3. Bounding Box

Keypoints belonging to the object box were assigned the class index, and keypoints outside the box were assigned the background index. After generating the class label, which was the index of the class, the label of the box to be predicted was also created. The box consisted of center coordinates ($x_{c}$, $y_{c}$, $z_{c}$), width, depth, and height information, indicating the size of the box, and values indicating the pitch, roll, and yaw of the rotation degree of the box. Pitch, roll, and yaw were the values converted from quaternion rotation. The nine values were converted into box labels, corresponding to the box to which the keypoint belonged, to be used as the predicted ground truth label.

As the center coordinates reflected the relative information of the coordinates when the relative adjacency matrix was applied, a vector value, calculated through the difference between the center of the box and the keypoint, was used. We referred to [17] and, for normalization, the width, depth, and height of the box were divided by the standard box size representing the object, and the log was then taken and normalized to obtain a value between 0 and 1. In this case, the size of the reference box used the average value of the object class size. The created class and box labels were used by the learner. In box decoding, because the value predicted through learning was normalized through box encoding, the previous process was performed in reverse order and converted into information corresponding to the original point cloud. In addition, because a box was predicted for each keypoint, if the same object was predicted, it was converted into one box through NMS.

## 4. Experimental Evaluation

### 4.1. Experimental Setup

#### 4.1.1. Dataset

**Figure 7:**
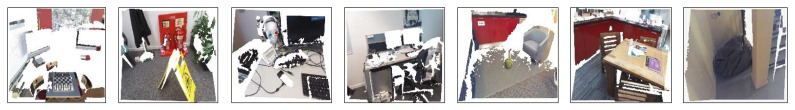
Objects selected for evaluation, include Chess, Fire, Heads, Office, Pumpkin, Red Kitchen, and Stairs.

In the study, we used the RGB-D Dataset 7-Scenes [32]. The dataset is a collection of tracked RGB-D camera frames and can be used for the evaluation of methods for different applications, such as dense tracking, mapping, and re-localization techniques. For our experiments, the dataset was converted into high-density 3D point cloud ply-format data using RGB and depth images, as shown in Figure 7. For the network performance experiment, several sequences were divided into separate training and test sets for each scene as a class.

#### 4.1.2. Implementation Details

Our architecture consisted of five core modules and the main unit that integrated these modules. The five modules were the data loader module to input data, the graph converter module to convert point cloud into the graph, preprocessing and normalization algorithms that converted the ground truth box into training data, the box manager module including the NMS algorithm, the GCN model module consisting of the GCN layer, and the visualizer module providing visualization. The main module integrated the five modules and implemented learning and visualization through options. The graph converter module extracted keypoints, based on voxels, and connected the extracted keypoints to form an adjacency matrix and feature matrix that indicated a connection relationship. The graph converter module was a random voxel keypoint selector that extracted voxel-based keypoints and an edge generator that created an edge list by connecting keypoints. It was composed of an adjacency matrix converter that converted the generated edge list into an adjacency matrix in the form of a sparse matrix and a feature matrix converter that created a feature matrix. Finally, the generated matrix was converted into a tensor that could be used in Pytorch, an open-source machine-learning library, through a sparse tensor converter. Our implementation was written in Python 3.7 using PyTorch for GPU computation. A desktop with Linux Ubuntu 16.04 LTS, Ryzen 9 3900X CPU, and RTX 2080 SUPER 8G GPU was used.

In our method, the reusability of the program source was increased through modularization according to each role, and the overall learning system was divided into a preprocessing unit and a learning unit. The preprocessor extracted keypoints, using voxels, from the point cloud for graph learning and generated an edge list that indicated the connection relationship between the keypoints. Based on the generated edge list, it was converted into an adjacency matrix in the form of a sparse matrix, and a matrix separate from the existing adjacency matrix was additionally configured to reflect the relative information between points, which were characteristic of the edges. This was the same as the existing adjacency matrix at a point representing only the connection relationship. After constructing an existing adjacency matrix, a relative feature between the central keypoint and adjacent keypoints was created when performing a matrix multiplication operation with a feature matrix. A relative adjacency matrix that reflected the number of edge connections was constructed in the loop part of the adjacency matrix. The characteristics of keypoints comprised a feature matrix, and the class index and box information were given to each keypoint for learning.

The processed adjacency matrix and feature matrix were used when entering the learning part and aggregating the features of adjacent points. To reflect the characteristics of the relative coordinates, our proposed KAT was used to reflect the relative characteristics between the keypoint and neighboring points through multiplication with the feature matrix. The newly created feature matrix was multiplied by the adjacency matrix to aggregate the features of first-order adjacency, and a wide range of features was aggregated through multiple iterations. The aggregated features were generalized through the read-out process, and the values were then estimated through the fully connected layer corresponding to the class and box pose. After learning had progressed sufficiently, when the saved model was loaded and the prediction was performed, the process that was normalized for learning was performed in reverse order for visualization. After converting the original information, the final prediction was made by removing the overlapping box using NMS.

We used three iterations in the GCN layer. The GCN layer performed The KAT and the skip connection. The size of each class was (64, 64, 9). We set $\left(l_{m},\;h_{m},\;w_{m}\right)$ as the median size of the bounding box including objects. Classes were predicted, along with the background and other classes. We set the point cloud as a point cloud down-sampled to a voxel size of 0.2 m. In the case of the initial vertex state, a multilayer perceptron (MLP) [32, 64, 128, 256] was used to include the raw point, and another MLP [256] was used in the Readout process. In NMS, threshold = 0.01 was set. We used stochastic gradient descent (SGD). We trained the network for 2K steps. Since the RGB-D Dataset 7-Scenes did not contain a specific validation set, we separated several blocks of 100 consecutive frames from the training data for use as validation data (10% per scene).

### 4.2. Results

**Figure 8:**
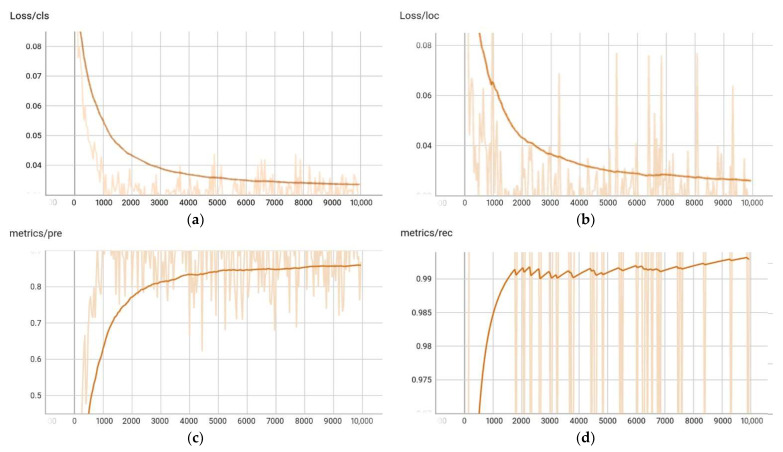
Metrics graph obtained from the GCN learning on the Chess in the 7-Scenes Dataset.; x-label (# of iteration), y-label ((**a**) class loss; (**b**) location loss; (**c**) precision; (**d**) recall.)

In the RGB-D Dataset 7-Scenes, we measured the IoU, and compared the center distances of the ground truth and predicted boxes in the same manner as in other competitive methods. In addition, the angles of the ground truth and predicted boxes were measured and compared. Figure 8 shows the learning loss of a representative class of object boxes estimated using the proposed method. This was also the amount of change in the classification performance evaluation index, location accuracy, precision, and recall.

Table 1 summarizes the results of the loss values obtained by learning the seven object types selected, based on our method. It was confirmed that high object recognition accuracy could be obtained during training.

**Figure 9:**
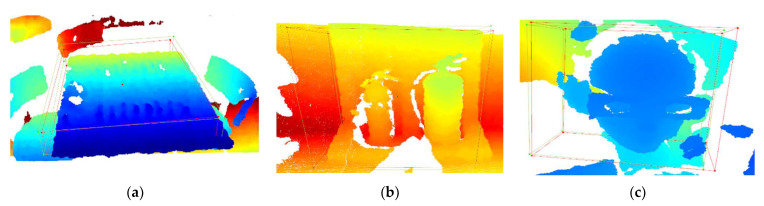
Visualize the pose estimation of objects in the 7-Scenes Dataset. The green box is the ground truth, red box is the prediction. (**a**) chess; (**b**) fire; (**c**) head.

As shown in Figure 9, the green box was the ground truth, and the red box was visualized by prediction. Table 2 shows the IoU comparison of the ground truth and prediction boxes for each class. We also measured the median pose error for all frames in the dataset. Our method was similar to the competing method in IoU of the poses of the ground truth box and the estimated box and showed that it could compete with other state-of-the-art methods in terms of accuracy, with an error of approximately 10 cm from the center of the box.

Table 3 compares our results on the 7-Scenes Dataset with those of the conventional methods. Pose (h, h*) = max ((θ, θ*), t − t*), where *h* is the estimated pose and h* is the true pose, *θ* is the axial angle representation of the camera rotation, and *t* is the camera translation. We measured the angle (*θ*, θ*) between the estimated and true rotations in degrees and the distance t − t* between the estimated and true transformations in cm.

## 5. Further Analyses

### 5.1. Keypoint Attention Mechanism Performance Comparison Analysis

We compared the object pose estimation accuracy with and without the KAT. Table 4 shows the accuracy of the network when the KAT was not used.

### 5.2. The GCN Module of Iterations

The GCN module of our method was composed of other GCN layers, including skip connection, to improve performance by iterations. In our experiment, the performance was degraded whenever iterating more than 4 times. Three iterations were determined by securing optimal performance and real-time process, as shown in Table 5 and Table 6.

### 5.3. Point Cloud Down-Sampling and Runtime Analysis

The speed of the pose-estimation algorithm is important for real-time applications. However, several factors affect the execution time, including algorithm architecture, code optimization, and hardware resources. Moreover, optimizing the implementation was not the focus of this study. However, the decomposition of the current inference time is helpful for future network optimizations. The hardware configuration used to measure the running time was that described in Section 4.1.2. We measured the inference time after learning the GCN by down-sampling 28K sparse points for each object in indoor space to speed up object pose estimation by reducing the number of calculations on the dense point cloud generated using the 7-Scenes Dataset. The average processing time for one sample in the validation was 43.2 ms. It took 77.38% of the time (33.45 ms) to read the dataset and run the calibration. Creating a graph representation required 16.82% of the time (7.27 ms). The inference of GCN took 1.51% of the time (0.65 ms). Box merging and scoring accounted for 3.03% of the total time (1.31 ms). The running time of the network was compared by down-sampling the point cloud through the voxels and expressing the down-sampled point cloud as a graph.

Table 6 compares the runtime after down-sampling to voxel sizes of 0.2 m and 0.4 m. We compared the accuracy owing to down-sampling and confirmed the optimized inference speed after down-sampling to a voxel size of 0.4 m.

## 6. Conclusions

In this work, we proposed the GCN for object detection and pose estimation in irregular point clouds represented by graphs.

Our method consisted of an end-to-end approach to class prediction and a box prediction and pose estimation by spatially structuring the graph. By aggregating the point features of the point cloud and its neighbors, the graph structure was reused in every process to increase the efficiency of memory usage. By designing a GCN model for efficient feature encapsulation, we improved the speed by simplifying the learning and prediction layers compared with GNN-based systems. Accuracy was also improved by introducing a relative adjacency matrix to reflect the relative characteristics of relative coordinates between neighboring points. In addition, owing to the low memory usage, learning and prediction was possible, not only on the GPU, but also on the CPU. Accuracy similar to that of the latest competitive methods was achieved through various preprocessing algorithms and skip-connection techniques. 

In a future study, to better reflect the original information when extracting keypoints through voxels, we will design a feature-impregnation layer for each voxel around the keypoints to achieve higher accuracy and represent regular inputs as graphs.

## Figures and Tables

**Table 1:** Loss of object pose estimation.

Loss	Chess	Fire	Heads	Office	Pumpkin	RedKitchen	Stairs
cls_loss	0.0060	0.0027	0.0310	0.0330	0.0300	0.0330	0.0340
loc_loss	0.0135	0.0100	0.0080	0.1580	0.0120	0.0390	0.0080
recall	0.8598	0.9286	1.0000	0.9752	0.8756	1.0000	1.0000
precision	0.9200	0.8667	0.8333	0.9100	0.9910	0.9518	0.9302

**Table 2:** IoU of the ground truth box and estimated box poses for each class.

Class	Chess	Fire	Heads	Office	Pumpkin	RedKitchen	Stairs
IoU	0.8719	0.9247	0.9118	0.8372	0.9112	0.8782	0.8782

**Table 3:** Median translation and rotation errors of our method with different pose estimators on the 7-Scenes dataset.

7-Scenes	Hourglass-Pose [33]	BranchNet [34]	VLocNet [35]	VLocNet++ [36]	RelocNet [37]	DSAC [38]	Ours
Chess	0.15 m, 6.53°	0.18 m, 5.17°	0.036 m, 1.71°	0.023 m, 1.44°	0.12 m, 4.14°	0.02 m, 1.2°	0.032 m, 1.44 °
Fire	0.27 m, 10.84°	0.34 m, 8.99°	0.039 m, 5.34°	0.018 m, 1.39°	0.26 m, 10.4°	0.04 m, 1.5°	0.021 m, 1.24°
Heads	0.19 m, 11.63°	0.20 m, 14.15°	0.046 m, 6.64°	0.016 m, 0.99°	0.14 m, 10.5°	0.03 m, 2.7°	0.021 m, 2.8 1°
Office	0.21 m, 8.48°	0.30 m, 7.05°	0.039 m, 1.95°	0.024 m, 1.14°	0.18 m, 5.32°	0.04 m, 1.6°	0.052 m, 1.78°
Pumpkin	0.25 m, 7.01°	0.27 m, 5.10°	0.037 m, 2.28°	0.024 m, 1.45°	0.26 m, 4.17°	0.05 m, 2.0°	0.028 m, 2.12°
RedKitchen	0.27 m, 10.84°	0.33 m, 7.40°	0.039 m, 2.20°	0.025 m, 2.27°	0.23 m, 5.08°	0.05 m, 2.0°	0.032 m, 2.53°
Stairs	0.29 m, 12.46°	0.38 m, 10.26°	0.097 m, 6.48°	0.021 m, 1.08°	0.28 m, 7.53°	1.17 m, 33.1°	0.031 m, 3.26°

**Table 4:** IoU values without the keypoint attention mechanism.

Not Used The KAT	Chess	Fire	Heads	Office	Pumpkin	RedKitchen	Stairs
IoU	0.4890	0.7708	0.8423	0.8936	0.7704	0.8935	0.7113

**Table 5:** Error angle and error distance by the number of iterations in the 7-Scenes Dataset.

GCN Layer	1st Iteration	2nd Iteration	3rd Iteration	4th Iteration
Error Distance	0.012 m	0.023 m	0.014 m	0.019 m
Error Angle	1.768°	1.562°	1.244°	1.400°

**Table 6:** Running time comparison after point cloud down-sampling through voxel size.

Running Time (s)	Chess	Fire	Heads	Office	Pumpkin	RedKitchen	Stairs
voxel size 0.2 m	0.0287	0.0490	0.0396	0.0507	0.0360	0.0486	0.0501
voxel size 0.4 m	0.0279	0.0472	0.0386	0.0447	0.0355	0.0452	0.0477

## Data Availability

The code and dataset will be made available upon request to the first author’s email with appropriate justification. The public site for each dataset was as follows: RGB-D Dataset 7-Scenes: https://www.microsoft.com/en-us/research/project/rgb-d-dataset-7-scenes/ (accessed on 25 October 2022).
